# Nonsteroidal anti-inflammatory drugs use and risk of Parkinson disease: A dose–response meta-analysis

**DOI:** 10.1097/MD.0000000000012172

**Published:** 2018-09-14

**Authors:** Li Ren, Jie Yi, Jing Yang, Peng Li, Xueyan Cheng, Peixian Mao

**Affiliations:** Department of Neurology, Beijing An Ding Hospital, Beijing, P. R. China.

**Keywords:** dose–response relationship, meta-analysis, NSAIDs, observational study, Parkinson disease

## Abstract

Previous studies have indicated that nonsteroidal anti-inflammatory drugs (NSAIDs) use is associated with Parkinson disease risk, but presented controversial results.

Medline, Embase, Web of Science, and the Cochrane Database were searched update to November 2017. Key data were extracted from eligible studies. A dose–response meta-analysis was conducted for synthesizing data from eligible studies.

Fifteen eligible studies were included in this meta-analysis. NSAIDs use was not associated with Parkinson disease risk [relevant risk (RR): 0.06; 95% confidence interval (95% CI), 0.91–1.02]. Subgroup analysis showed that aspirin use (RR: 1.14; 95% CI, 0.98–1.30) or ibuprofen use (RR: 1.01; 95% CI, 0.88–1.17) was not associated with Parkinson disease risk; however, the use of non-aspirin NSAIDs was significantly associated with Parkinson disease risk (RR:0.91; 95% CI, 0.84–0.99). Furthermore, NSAIDs use was not associated with the risk of Parkinson disease in female (RR: 0.99; 95% CI, 0.83–1.17) and male (RR: 1.01; 95% CI, 0.88–1.16). In addition, a dose–response showed per 1 number of prescription incremental increase in NSAIDs use was not associated with the risk of Parkinson disease (RR: 0.96; 95% CI, 0.91–1.02), per 1 year of duration of NSAIDs use incremental increase was not associated with the risk of Parkinson disease (RR: 0.98; 95% CI, 0.92–1.03), and per 1 dosage of NSAIDs use incremental increase was not associated with the risk of Parkinson disease (RR: 0.98; 95% CI, 0.95–1.02).

NSAIDs use was not associated with the risk of Parkinson disease. The potency and the cumulative NSAIDs use did not play critical roles.

## Abstract 2

Supplemental Digital Content is available in the text

## 1 Introduction

Commonly known as nonsteroidal anti-inflammatory drugs (NSAIDs), they are widely used to eliminate pain, swelling, stiffness, and inflammation of the limbs, and they have been widely used in clinically.^[1]^ In addition to anti-inflammatory, NSAIDs gradually attracts people's attention in the prevention and treatment of Parkinson disease.^[2]^ NSAIDs use is correlated with Parkinson disease and is biologically understandable. Neuroinflammation was correlated with the pathogenesis of Parkinson disease, and NSAIDs provide neuroprotection in animal models.^[3,4]^

It is estimated that there are more than 1 billion NSAIDs prescriptions in the world every year, and about 30 million people take NSAIDs every day.^[1]^ The annual prescription of NSAIDs in the United States is about 70 million, accounting for 4% of all drug prescriptions.^[5,6]^ In the UK, the prescription volume of NSAIDs is about 20 million, and 15% of the people over the age of 60 take NSAIDs.^[7]^ Australia has about 11 million NSAIDs prescriptions per year, accounting for 5% of all drug prescriptions.^[1]^ NSAIDs have been preliminarily proven to reduce the incidence of Alzheimer disease, which makes the application of NSAIDs uptrend.^[8]^ Although NSAIDs use has a potential to prevent Parkinson disease, there have been safety concerns about their impact on Parkinson disease.^[9,10]^ Currently, there are continued concerns, partly due to the conflicting results of the association between NSAIDs use and Parkinson disease.

Considering increasing number of patients being prescribed NSAIDs use, clinicians, pharmacists, patients, society, and governments pay more attention to the safety of these drugs. We conducted a meta-analysis based on observational studies to determine whether NSAIDs use is associated with Parkinson disease risk.

## 2 Methods

There are no ethical issues involved in our study for our data were based on published studies.

### 2.1 Search strategy

Eligible studies were systematically searched of Medline, Embase, Web of Science, and the Cochrane Database were searched update to November 2017 examining the association between NSAIDs use and Parkinson disease risk, with keywords, including “Parkinson Disease” [MeSH] or “PD” [MeSH] or “Lewy Body Parkinson's disease” [MeSH] or “Idiopathic Parkinson's Disease” [MeSH] and “NSAIDs” [MeSH] or “Non-Steroidal Anti-Inflammatory Agents” [MeSH] or “Anti-Inflammatory Analgesics” [MeSH] or “nonsteroidal anti-inflammatory drugs” [MeSH].

### 2.2 Inclusion and exclusion criteria

Investigators independently collect information: first, the outcome was Parkinson disease; second, risk estimates on the relationship between NSAIDs use and Parkinson disease risk. According to the Newcastle–Ottawa scale, quality assessment was performed for nonrandomized studies.^[11]^

### 2.3 Statistical analysis

Due to different definitions of cut-off points in the included studies for categories, we performed a relative risk estimates by the method recommended by Orsini et al.^[12]^ In addition, use restricted cubic splines (RCS) to evaluate the nonlinear association between NSAIDs use and Parkinson disease risk, with 3 knots at the 10th, 50th, and 90th percentiles of the distribution. A flexible meta-regression based on RCS function was used to fit the potential nonlinear trend, and generalized least-square method was used to estimate the parameters. This procedure treats NSAIDs use (continuous data) as an independent variable and logRR of diseases as a dependent variable, with both tails of the curve restricted to linear. A *P* value is calculated for linear or nonlinear by testing the null hypothesis that the coefficient of the second spline is equal to zero.

We use STATA software 14.0 (STATA Corp, College Station, TX) to evaluate the relationships between NSAIDs use and Parkinson disease risk. Heterogeneity among studies used Q test and *I*^2^ statistic to assess. If *P*_Q_ < .10 or *I*^2^ > 50%, random-effect model was chosen; otherwise, fixed-effect mode was applied. Begg and Egger tests were used to assess the publication bias of each study. *P* < .05 was considered significant for all tests.

## 3 Results

### 3.1 Literature search results

**Figure 1:**
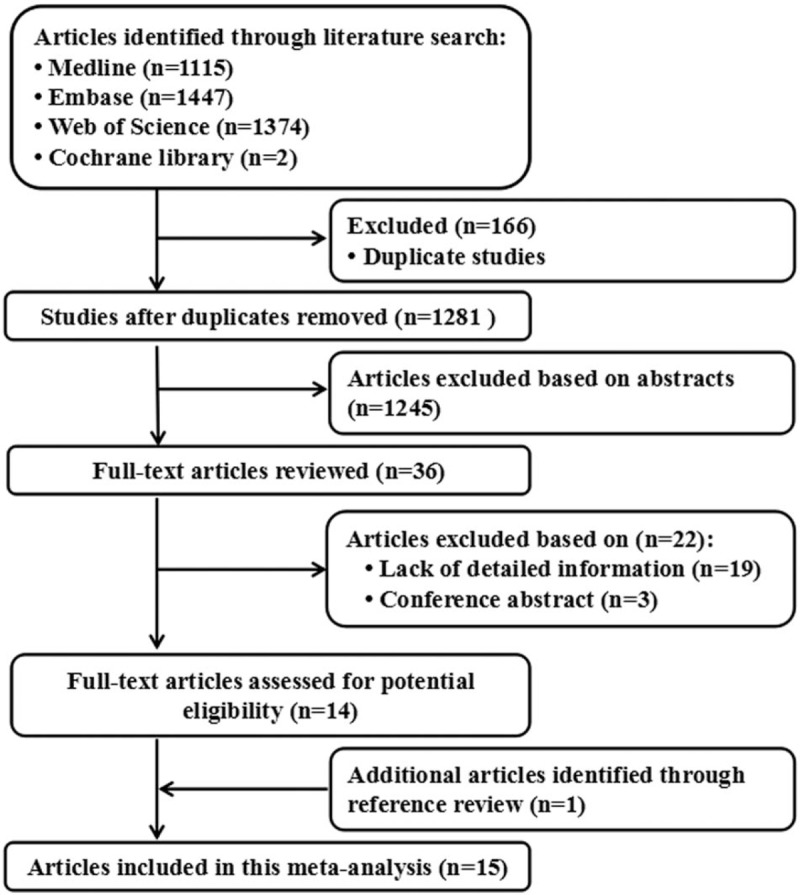
Flow diagram of the study selection process.

A total of 1115 studies from Medline, 1447 studies from Embase, and 1374 studies from Web of Science were included. After removing duplicates study, 1281 studies were identified. Reviewing their titles and abstracts, 1245 citations were excluded for no relevant outcome or nonhuman studies or reviews. The remaining 36 citations were assessed in more detail for eligibility by reading the full text. Among them, 19 studies were excluded due to lack of detailed information; 3 studies were excluded due to conference abstract. After review reference of studies, 1 article was identified. Finally, 15 studies were used for the final data synthesis.^[13–27]^ The flow chart of literature searching is presented in Fig. 1. The characteristics of the included studies are summarized in the Tables 1 and 2 .

**Table 1:**
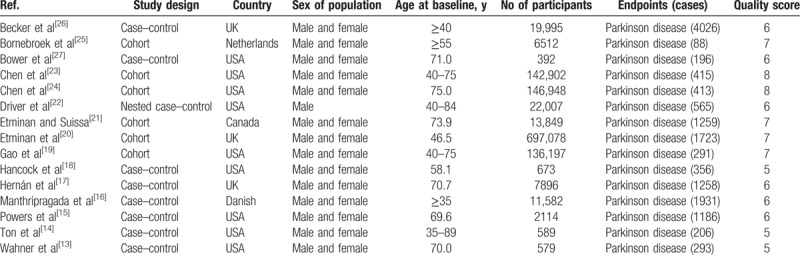
Characteristics of participants in included studies of NSAIDs use in relation to risk of Parkinson disease.

**Table 2:**
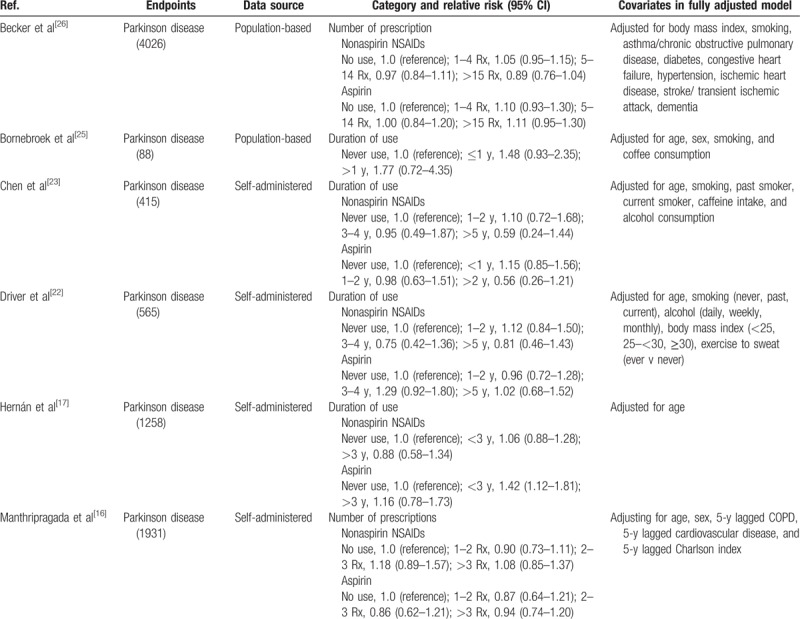
Outcomes and covariates of included studies of NSAIDs use in relation to risk of Parkinson disease.

**Table 2 (Continued):**
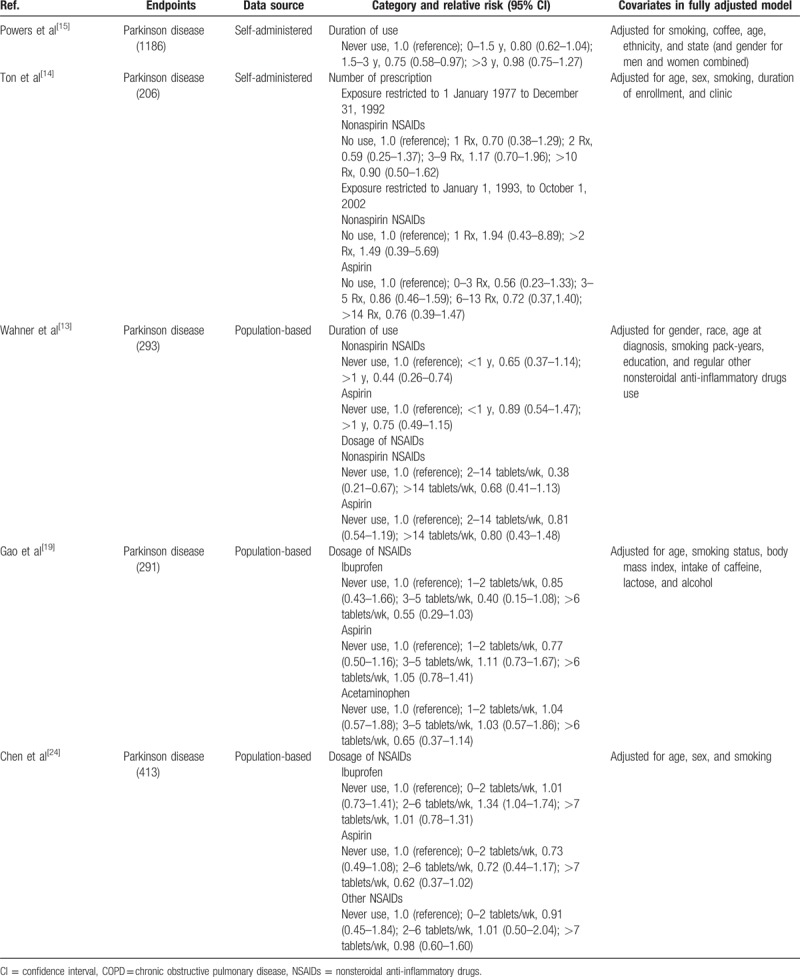
Outcomes and covariates of included studies of NSAIDs use in relation to risk of Parkinson disease.

### 3.2 NSAIDs use and Parkinson disease risk

Fifteen eligible studies including 25 independent reports identified the relationship between NSAIDs use and Parkinson disease risk. NSAIDs use was not associated with Parkinson disease risk [relevant risk (RR): 0.96; 95% confidence interval (95% CI), 0.91–1.02; *P* = .174] (Table 3). Subgroup analysis showed that aspirin use (RR: 1.14; 95% CI, 0.98–1.30; *P* = .467) (Table 3) or ibuprofen use (RR: 1.01; 95% CI, 0.88–1.17; *P* = .204) (Table 3) was not associated with Parkinson disease risk; however, nonaspirin NSAIDs use was significantly associated with Parkinson disease risk decrement (RR: 0.91; 95% CI, 0.84–0.99; *P* = .028) (Table 3). Furthermore, NSAIDs use was not associated with Parkinson disease risk in female (RR: 0.99; 95% CI, 0.83–1.17; *P* = .876) (Table 3) and male (RR: 1.01; 95% CI, 0.88–1.16; *P* = .913) (Table 3).

**Table 3:**
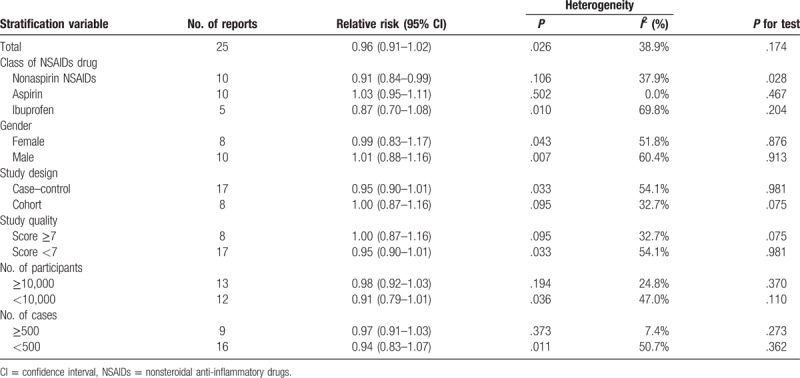
Stratified analysis of relative risk of Parkinson disease.

### 3.3 Dose–response between NSAIDs use and risk of Parkinson disease

**Figure 2:**
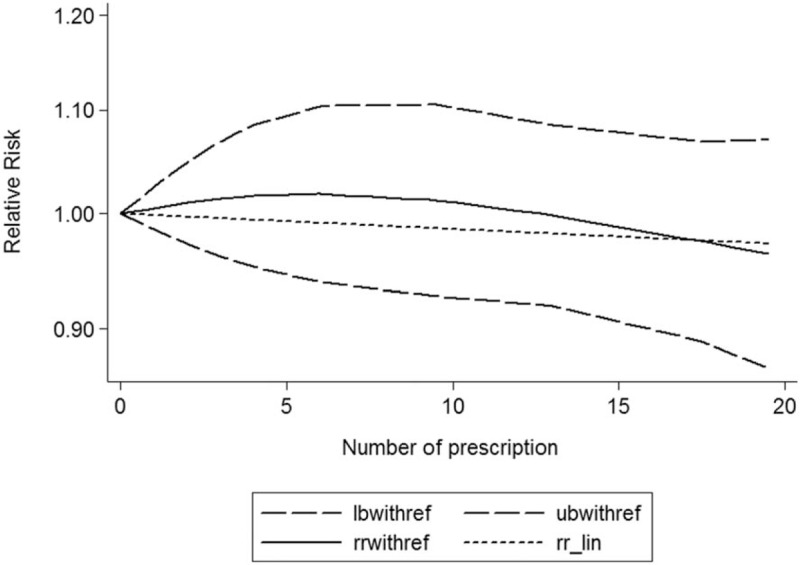
Dose–response relationship between number of prescription NSAIDs use in relation to risk of Parkinson disease.

**Figure 3:**
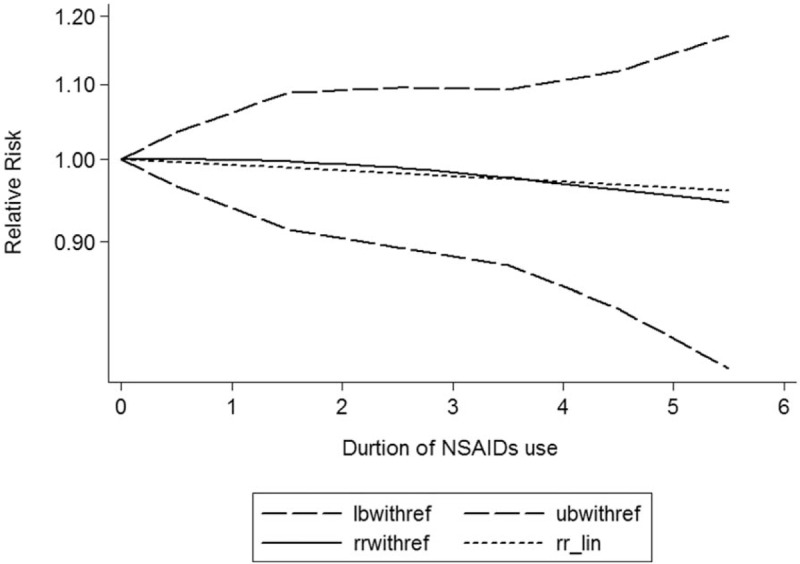
Dose–response relationship between duration of NSAIDs use in relation to risk of Parkinson disease.

**Figure 4:**
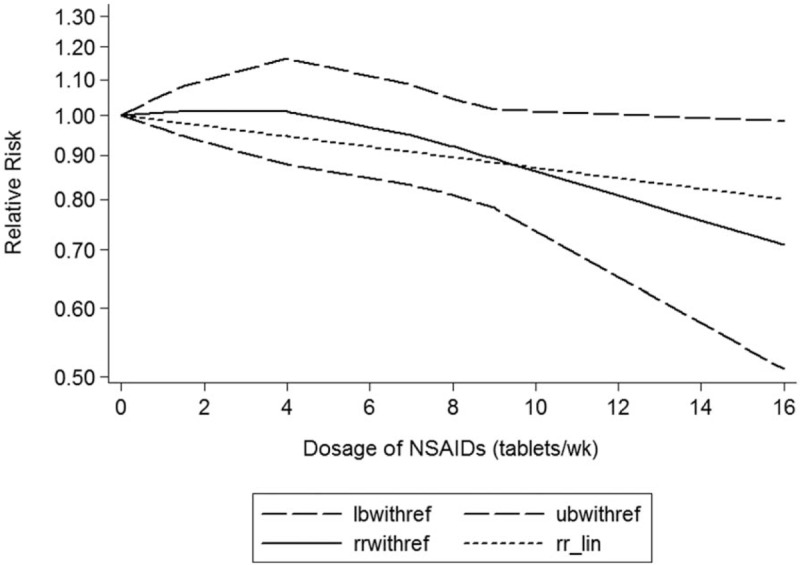
Dose–response relationship between dosage of NSAIDs use in relation to risk of Parkinson disease.

A dose–response showed that per 1 number of prescription incremental increase in NSAIDs use was not associated with Parkinson disease risk (RR: 0.96; 95% CI, 0.91–1.02; *P* = .513) (Fig. 2), per 1 year of duration of NSAIDs use incremental increase was not associated with Parkinson disease risk (RR: 0.98; 95% CI, 0.92–1.03; *P* = .404) (Fig. 3), and per 1 dosage of NSAIDs use incremental increase was not associated with Parkinson disease risk (RR: 0.98; 95% CI, 0.95–1.02; *P* = .103) (Fig. 4).

### 3.4 Publication bias

Statistical tests suggest that there was no evidence of publication bias [Begg test (*P* = .44) and Egger test (*P* = .56)] (Supplementary table 1). A funnel plot for publication bias assessment is illustrated in supplementary figure 1 to 3.

## 4 Discussion

Parkinson disease, also known as tremor paralysis, is one of the most common neurodegenerative diseases, and slow motion, myotonia, tremor, abnormal gait, cognitive disorders, sleep disorders, autonomic dysfunction, and sensory disorders are the main features of this disease.^[28,29]^ There is no specific treatment for Parkinson disease.^[30]^ The treatment of Parkinson disease is mainly from reducing the pain, delaying the development of the disease, and improving the quality of life of the patients. Although some of the drugs (such as cholinesterase inhibitors) can improve the ability of patients to accept new things in short term and delay the aggravation of Parkinson disease, the long-term curative effect remains to be observed. Antipsychotic drugs can be used to combat psychotic symptoms, agitation, or aggressive behavior.^[31]^ Antidepressants can be used in patients with dementia and depression, and help to improve the dementia syndrome. But it must be noted that the anticholinergic side effects of tricyclic drugs can aggravate cognitive impairment.^[32]^ Although benzodiazepines use can control the behavior problem of the Parkinson disease, it should be specially cautious because it can cause falls and drug dependence.^[33]^ These drugs in the treatment of Parkinson disease more or less have some kinds of problems, and NSAIDs have been widely used in anti-inflammatory therapy, and its pleiotropic effects have expanded its clinical value and the potential therapeutic effect of NSAIDs on Parkinson disease is expected.

NSAIDs are a class of drugs that eliminate prostaglandin synthetase to eliminate inflammation. A variety of evidence suggests that inflammation contributes to the pathogenesis of Parkinson disease; NSAIDs have a potential therapeutic effect on Parkinson disease, although it has an anti-inflammatory effect.^[34,35]^ However, the relationship between NSAIDS use and Parkinson disease is inconclusive. Among these selected studies, most of the reports have found NSAIDs use was not associated with Parkinson disease, but one report found that NSAIDs use was associated with Parkinson disease risk decrement.^[13]^ Two reports found nonaspirin NSAIDs was associated with Parkinson disease risk decrement,^[13,23]^ and either no effect or increased Parkinson disease risk. Aspirin use was not associated with a decreased risk of Parkinson disease in all studies. Ibuprofen use was slightly associated with Parkinson disease risk decrement in 2 studies.^[13,16]^

Two meta-analyses have identified the relationship between NSAID use and Parkinson disease risk, but presented controversial results. Gagne and Power,^[36]^ based on seven observational studies, found nonaspirin NSAIDs use was associated with Parkinson disease risk decrement (RR: 0.85; 95% CI, 0.77–0.94), and aspirin was not associated with Parkinson disease risk (RR: 1.08; 95% CI, 0.92–1.27). However, Samii et al,^[37]^ based on 11 observational studies, found NSAIDs use (RR: 0.95; 95% CI, 0.80–1.12) and aspirin (RR: 1.08; 95% CI, 0.93–1.26) was not associated with risk of Parkinson disease, but ibuprofen use was slightly associated with Parkinson disease risk decrement (RR: 0.76; 95% CI, 0.65–0.89). Also, Samii et al^[37]^ found that NSAIDs use was associated with Parkinson disease risk decrement in male (RR: 0.79; 95% CI, 0.69–0.92) but not female (RR: 0.72; 95% CI, 0.45–1.15).

Considering these conflicting results, this meta-analysis was based on the latest evidence update to November 2017 from 15 studies supporting that NSAIDs use was not associated with the risk of Parkinson disease, and a dose–response showed per 1 number of prescription incremental increase in NSAIDs use was not associated with risk of Parkinson disease (RR: 0.96; 95% CI, 0.91–1.02), per 1 year of duration of NSAIDs use incremental increase was not associated with risk of Parkinson disease (RR: 0.98; 95% CI, 0.92–1.03), and per 1 dosage of NSAIDs use incremental increase was not associated with risk of Parkinson disease (RR: 0.98; 95% CI, 0.95–1.02); the potency and the cumulative NSAIDs use did not play critical roles. This meta-analysis included enough studies; these results should be credible.

This meta-analysis also has some limitations. First, we have never tried to search for unpublished research, which may lead to the disappearance of related research. Second, we did not include randomized controlled trials due to Parkinson disease, which was not a prespecified endpoint in randomized controlled trials; on the contrary, test results of the NSAIDs use statistical heterogeneity were limited in randomized controlled trials, and limited evidence of a dose-dependent association between NSAIDs and Parkinson disease risk provides limited confidence in their findings, and randomized controlled trials should be included in further studies.

This meta-analysis indicates that that NSAIDs use was not associated with risk of Parkinson disease. The potency and the cumulative NSAIDs use did not play critical roles. In the future, large-scale and population-based association studies must be performed in the future to validate the risk identified in the current meta-analysis.

## Author contributions

**Data curation:** Li Ren, Jie Yi, Xueyan Cheng, Peixian Mao.

**Formal analysis:** Jie Yi, Peixian Mao.

**Investigation:** Peng Li, Peixian Mao.

**Project administration:** Peng Li.

**Software:** Li Ren, Jing Yang, Peixian Mao.

**Writing – original draft:** Peixian Mao.

**Writing – review & editing:** Peixian Mao.

## Supplementary Material

Supplemental Digital Content
