# Supplementary material for: Nonsteroidal anti-inflammatory drugs use and risk of Parkinson disease: A dose–response meta-analysis
Source: Medicine (Baltimore). 2018 Sep 14;97(37):e12172. doi: 10.1097/MD.0000000000012172 (PMC6155958; doi:10.1097/MD.0000000000012172)

**Supplementary table 1. Publication bias analysis of the meta-analysis**

|  | Test | t | 95% CI | P |
| --- | --- | --- | --- | --- |
| NSAIDs use and Parkinson's disease risk | Begg’s test |  |  | 0.438 |
|  | Egger’s test | 0.38 | -0.15,0.92 | 0.561 |
| Non-aspirin NSAIDs use and Parkinson's disease risk | Begg’s test |  |  | 0.631 |
|  | Egger’s test | 0.93 | -1.86,1.97 | 1.000 |
| Aspirin use and Parkinson's disease risk | Begg’s test |  |  | 0.253 |
|  | Egger’s test | 0.97 | -1.13,1.89 | 0.841 |

**Supplementary figure 1: A funnel plwot for the meta-analysis of NSAIDs use and Parkinson’s disease risk**

**
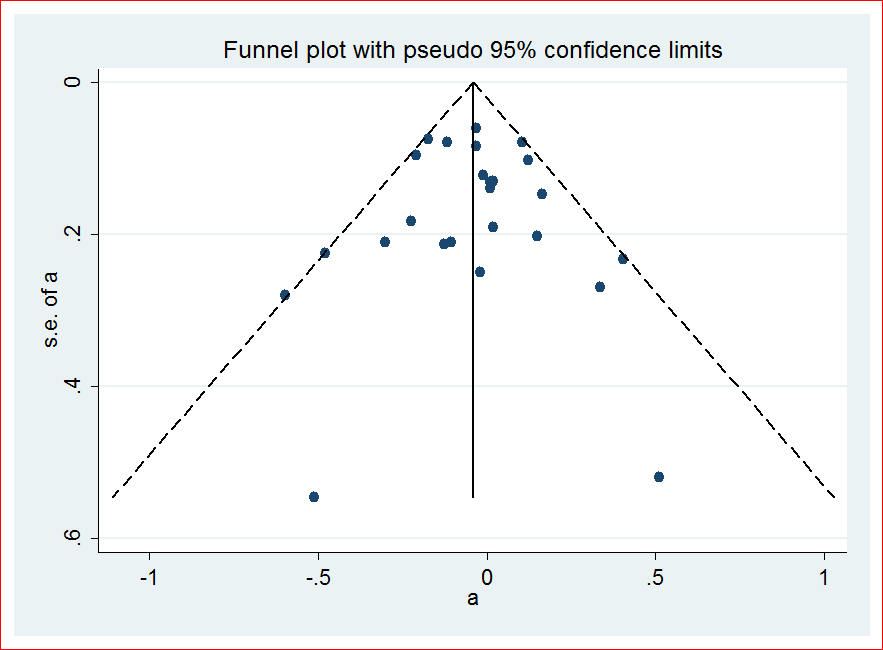
**

**Supplementary figure 2: A funnel plot for the meta-analysis of non-aspirin NSAIDs use and Parkinson’s disease risk**

**
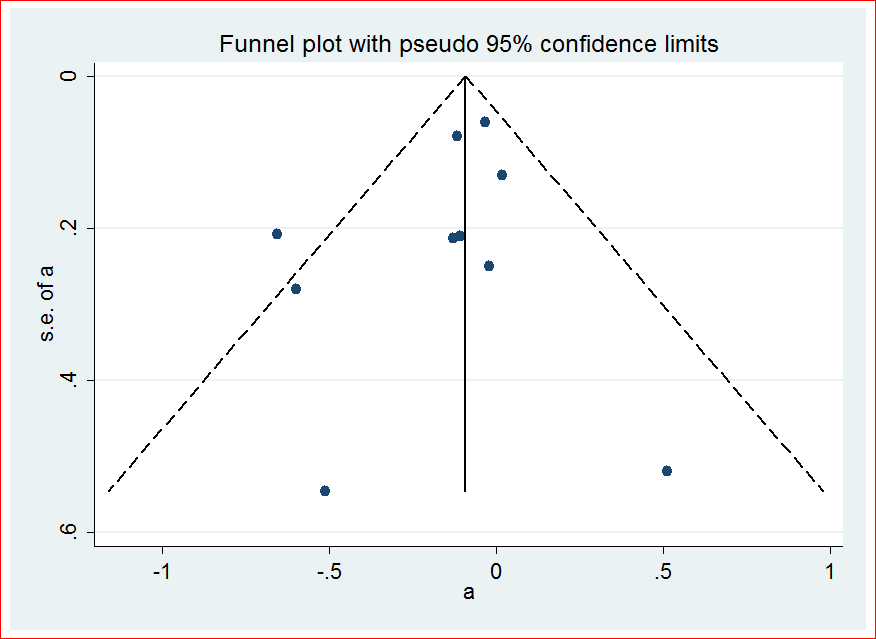
**

**Supplementary figure 3: A funnel plot for the meta-analysis of aspirin use and Parkinson’s disease risk**


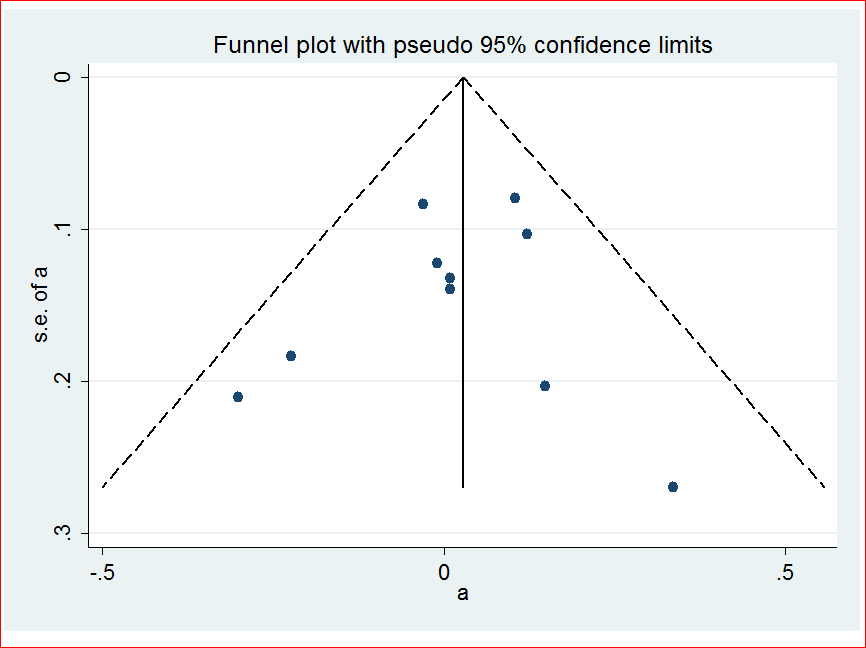

Supplement: Supplemental Digital Content [file medi-97-e12172-s001.docx]
